# Dataset of 3D gait analysis in typically developing children walking at three different speeds on an instrumented treadmill in virtual reality

**DOI:** 10.1016/j.dib.2023.109142

**Published:** 2023-04-12

**Authors:** Rachel Senden, Rik Marcellis, Kenneth Meijer, Paul Willems, Ton Lenssen, Heleen Staal, Yvonne Janssen, Vincent Groen, Roland Jeroen Vermeulen, Marianne Witlox

**Affiliations:** a Department of Physical Therapy, Maastricht University Medical Center, Postbus 5800 AZ, Maastricht 6020, the Netherlands; b Department of Nutrition and Movement Sciences, NUTRIM School of Nutrition and Translational Research in Metabolism, Maastricht University, Maastricht, the Netherlands; c Department of Orthopaedic Surgery, Maastricht University Medical Center, Maastricht, the Netherlands; d Research School CAPHRI, Maastricht University, Maastricht, the Netherlands; e Centre of Expertise in Rehabilitation and Audiology, Adelante, Hoensbroek, the Netherlands; f Department of Rehabilitation Medicine, School for Public Health and Primary Care, Maastricht University, Maastricht, the Netherlands; g Department of Neurology, Maastricht University Medical Center, Maastricht, the Netherlands

**Keywords:** Gait analysis, Typically developing children, CAREN, Spatiotemporal parameters, Joint angles, Ground reaction forces, Joint moments, Joint powers

## Abstract

In this article, gait data of typically developing (TD) children (24 boys/31 girls, mean (95% confidence interval) age 9.38 (8.51 – 10.25) years, body mass 35.67 (31.40 – 39.94) kg, leg length 0.73 (0.70 – 0.76) m, and height 1.41 (1.35 – 1.46) m) walking at different walking speeds is shared publicly.

Raw and processed data is presented for each child separately and includes data of each single step of both legs. Beside, the subject demographics and the results from the physical examination are presented allowing to select TD children from the database to create a matched group, based on specific parameters (e.g. sex and body weight).

For clinical application, gait data is also presented per age group, which provides quick insight into the normal gait pattern of TD children of varying age.

Gait analysis was performed during treadmill walking in a virtual environment using the Computer Assisted Rehabilitation Environment (CAREN). The human body lower limb model with trunk markers (HBM2) was used as biomechanical model. Children walked at comfortable walking speed, 30% slower and 30% faster (random sequence) while wearing gymnastic shoes and a safety harness to prevent falling. For each speed condition, 250 steps were recorded. Data quality check, step detection and the calculation of gait parameters was done by custom made Matlab algorithms.

Raw data files are provided per walking speed, for each child separately. The raw data is exported from the CAREN software (D-flow) and is provided in .mox and .txt files. It includes the output from the models such as subject data, marker and force data, kinematic data (joint angles), kinetic data (joint moments, GRFs, joint powers), as well as CoM data and EMG data (the last two are not described in this manuscript), for each speed condition and each child. Unfiltered and filtered data are included. C3D files with raw marker and GRF data were recorded in Nexus (Vicon software) and are available upon request.

After analyzing the raw data into Matlab (R2016a, Mathworks) using custom made Matlab algorithms, processed data is obtained. The processed data is provided in .xls files and is also presented for each child separately. It contains spatiotemporal parameters, 3D joint angles, anterior-posterior and vertical ground reaction forces (GRF), 3D joint moments and sagittal joint power of each step of the left and right leg.

In addition to each individual's data, overview files (.xls) are created per walking speed condition. These overviews present the averaged gait parameter (e.g. joint angle), calculated over all valid steps, of each child.

## Abstract (author highlights)

For clinical use, the processed data is also presented and visualized per age group (3-6 years, 7-8 years, 9-10 years, 11-12 years, ≥13 years). For each speed condition, spatiotemporal parameters, joint angles, joint moments and joint power for both legs are presented per age group. These data are presented in tables and visualized in waveforms and can serve as normative values for treadmill based 3D gait analyses.

Data is available at OSF.io (DOI 10.17605/OSF.IO/3XQEW).

This dataset can be used: (1) to assess walking biomechanics in typically developing children, (2) to assess walking biomechanics at various walking speeds, (3) to investigate relations between demographics and walking biomechanics, (4) as normative data to compare pathological gait with, and (5) investigating interchangeability of gait analysis approaches.

**Specifications Table**

**Table:** 

Subject	Walking biomechanics.
Specific subject area	3D gait analysis in typically developing children aged 3 to 17 years walking at three different walking speeds during treadmill walking in virtual environment.
Type of data	**Raw data**: .mox and .txt files are presented for each child individually and for each speed condition. Raw data includes the output of the biomechanical model like subject data, marker and force data, kinematic data (joint angles), kinetic data (joint moment, GRF, joint power), CoM data and EMG data (the last two are not described in this manuscript) of each single step of both legs. Unfiltered and filtered data are included. **Processed data:** .xls files are presented for each child individually and for each speed condition. Processed data includes spatiotemporal parameters, 3D joint angles, anterior-posterior and vertical GRF, 3D joint moments, sagittal joint power of each single step of both legs.
	For clinical application, gait data is also presented per age group (3-6y, 7-8y, 9-10y, 11-12y, >13y). **Figures:** Joint angles, GRF, joint moments and joint power are visualized in waveforms per age group (JPN figures), for each walking speed separately. **Tables:** Spatiotemporal parameters (average of left and right leg) are presented per age group in a table (JPN figures), for each walking speed separately.
How the data were acquired	The Computer Assisted Rehabilitation ENvironment (CAREN, Motek Medical BV) comprising an instrumented split-belt treadmill (ForceLink) integrated on a movable platform and surrounded by a 12-camera 3D motion capture system (Vicon Nexus v2.7) and a 180° cylindrical screen with virtual industrial environment, is used. The Human Body Model lower limb marker model (HBM2) is used as biomechanical model. Children walked 250 steps at comfortable (conform overground walking), slow (30% slower) and fast (30% faster) speed. The hardware components of CAREN are controlled by the Motek D-Flow software (V3.28.0). The 3D motion capture system is controlled by Vicon Nexus software (V2.8.1). Custom made Matlab algorithms (R2016a, Mathworks) were used for data quality check, step detection and the calculation of gait parameters.
Data format	Raw Filtered Analyzed
Description of data collection	55 TD children (3-17yrs) were included. Measurements were done at comfortable, slow and fast walking speed. Spatiotemporal and kine(ma)tic data of each single step of both legs is measured and presented for each child separately. Kine(ma)tic data were extrapolated to strides (0-100%). The raw data of each step of each child are presented in .mox and .txt files. The processed data of each step of each child are presented .xls files. This was done per walking speed condition. In addition, for each child the averaged gait pattern is determined by calculating the averages over all valid strides for every gait parameter. The averaged gait pattern of each child is presented in the overview files (.xls) Beside, data is categorized and presented in age groups: 3-6yrs (*n* = 11), 7-8yrs (*n* = 10), 9-10yrs (*n* = 15), 11-12yrs (*n* = 11), >13yrs (*n* = 8). This was done for each walking speed separately. GRFs were normalized for weight.
Data source location	• Institution: Maastricht University Medical Centre • City/Town/Region: Maastricht • Country: The Netherlands
Data accessibility	Repository name: OSF.io Direct URL to data: https://doi.org/10.17605/OSF.IO/3XQEW
Related research article	R. Senden, R. Marcellis, K. Meijer, P. Willems, T. Lenssen, H. Staal, Y. Janssen, V. Groen, R.J. Vermeulen, M. Witlox. Comparison of sagittal plane gait characteristics between the overground and treadmill approach for gait analysis in typically developing children. *PeerJ2022.* 10:e13752 https://doi.org/10.7717/peerj.13752[1].

## Value of the Data

• The measurement of many successive strides (250 steps recorded in current measurements) can contribute to the understanding of biomechanics of treadmill walking in typically developing children.
• The data can assist in improving the understanding of speed differences in walking biomechanics.
• Presenting each child's data separately, along with the subject demographics and the results from the physical examination, allows to create an own matched database This normative data can be used for comparison of pathological gait, thereby improving the interpretation of pathological gait and finally contributing to better clinical decision making.
• The data can be used to investigate explicit hypotheses such as studying associations between walking biomechanics and demographics (e.g. age, sex) at different walking speeds in typically developing children.
• As many successive strides are measured (250 steps were recorded in current measurements), the dataset allows to investigate gait variability and dynamic stability.
• The data can be useful for any scientist interested in gait and can be used for any scientific purposes in case permission is granted.

## 1 Objective

To create a database for treadmill walking in a virtual reality at various walking speeds in typically developing children.

## 2 Data Description

**File 01_Demo_PhysEx.xlsx:** contains each child's demographic data including age (years), sex (M/F), body mass (kg), height (m), leg length (m) and age group (1 = 3–6 years, 2 = 7–8 years, 3 = 9–10 years, 4 = 11–12 years, 5 = ≥13 year). In addition the results from the physical examination are presented per child.
**File 02_Overview_comf.xlsx:** contains the averaged walking biomechanics (calculated over all valid strides) for each child for walking at comfortable speed. Spatiotemporal parameters, joint angles, GRF, joint moments, and joint power are included for the left and right leg. This data is based on the individual excel files that can be found in “Folder 25_xls files”. The description of the parameters can be found in File 26_Description_parameters.
**File 03_ Overview_fast.xlsx:** contains the averaged walking biomechanics (calculated over all valid strides) for each child for walking at fast speed. Spatiotemporal parameters, joint angles, GRF, joint moments, and joint power are included for the left and right leg. This data is based on the individual excel files that can be found in “Folder 25_xls files”. The description of the parameters can be found in File 26_Description_parameters.
**File 04_ Overview_slow.xlsx:** contains the averaged walking biomechanics (calculated over all valid strides) for each child for walking at slow speed. Spatiotemporal parameters, joint angles, GRF, joint moments, and joint power are included for the left and right leg. This data is based on the individual excel files that can be found in “Folder 25_xls files”. The description of the parameters can be found in File 26_Description_parameters.
**File 05_AgeGroups_comf.xlsx**: contains walking biomechanics categorized per age group (3–6 year, 7–8 year, 9–10 year, 11–12 year, >13 years for walking at comfortable speed). Group averages and standard deviations for spatiotemporal parameters, joint angles, GRF, joint moments, and joint power are included for the left and right leg. The description of the parameters can be found in File 26_Description_parameters.
**File 06_AgeGroups_fast.xlsx**: contains walking biomechanics categorized per age group (3–6 year, 7–8 year, 9–10 year, 11–12 year, >13 years for walking at comfortable speed). Group averages and standard deviations for spatiotemporal parameters, joint angles, GRF, joint moments, and joint power are included for the left and right leg. The description of the parameters can be found in File 26_Description_parameters.
**File 07_AgeGroups_slow.xlsx**: contains walking biomechanics categorized per age group (3–6 year, 7–8 year, 9–10 year, 11–12 year, >13 years for walking at comfortable speed). Group averages and standard deviations for spatiotemporal parameters, joint angles, GRF, joint moments, and joint power are included for the left and right leg. The description of the parameters can be found in File 26_Description_parameters.
**File 08_Table_Spatiotemporal_comf.png:** represents spatiotemporal parameters per age group (3–6 year, 7–8 year, 9–10 year, 11–12 year, >13 years), including means and standard deviations for walking at comfortable speed. These spatiotemporal parameters are calculated using the private custom made algorithms (not the one calculated by D-flow).
**File 09_Fig_JointAngles_comf.png**: represents the averaged 3D joint angle waveforms of the right leg per age group (3–6 year, 7–8 year, 9–10 year, 11–12 year, >13 years) for walking at comfortable speed.
**File 10_Fig_GRF_comf.png:** represents the averaged vertical and anterior posterior GRF waveforms of the right leg per age group (3–6 year, 7–8 year, 9–10 year, 11–12 year, >13 years) for walking at comfortable speed. GRFs were normalized for body weight according to Hof [2].
**File 11_Fig_JointMoments_comf.png:** represents the averaged 3D joint moment waveforms of the right leg per age group (3–6 year, 7–8 year, 9–10 year, 11–12 year, >13 years) for walking at comfortable speed.
**File 12_Fig_JointPower_comf.png:** represents the averaged sagittal joint power waveforms of right leg per age group (3–6 year, 7–8 year, 9–10 year, 11–12 year, >13 years) for walking at comfortable speed.
**File 13_Table_Spatiotemporal_fast.png:** represents spatiotemporal parameters per age group (3–6 year, 7–8 year, 9–10 year, 11–12 year, >13 years), including means and standard deviations, for walking at fast speed. These spatiotemporal parameters are calculated using the private custom made algorithms (not the one calculated by D-flow).
**File 14_Fig_JointAngles_fast.png:** represents the averaged 3D joint angles waveforms of right leg per age group (3–6 year, 7–8 year, 9–10 year, 11–12 year, >13 years) for walking at slow speed.
**File 15_Fig_GRF_fast.png:** represents the averaged vertical and anterior-posterior GRF waveforms of right leg per age group (3–6 year, 7–8 year, 9–10 year, 11–12 year, >13 years) for walking at fast speed. GRFs were normalized for body weight according to Hof [2].
**File 16_Fig_JointMoments_fast.png:** represents the averaged 3D joint moments waveforms of right leg per age group (3–6 year, 7–8 year, 9–10 year, 11–12 year, >13 years) for walking at fast speed.
**File 17_Fig_JointPower_fast.png:** represents the averaged sagittal joint power waveforms of right leg per age group (3–6 year, 7–8 year, 9–10 year, 11–12 year, >13 years) for walking at fast speed.
**File 18_Table_Spatiotemporal_slow.png:** represents spatiotemporal parameters per age group (3–6 year, 7–8 year, 9–10 year, 11–12 year, >13 years), including means and standard deviations for walking at slow speed. These spatiotemporal parameters are calculated using the private custom made algorithms (not the one calculated by D-flow).
**File 19_Fig_JointAngles_ slow.png:** represents the averaged 3D joint angles waveforms of right leg per age group (3–6 year, 7–8 year, 9–10 year, 11–12 year, >13 years) for walking at slow speed.
**File 20_Fig_GRF_ slow.png:** represents the averaged vertical and anterior-posterior GRF waveforms of right leg per age group (3–6 year, 7–8 year, 9–10 year, 11–12 year, >13 years) for walking at slow speed. GRFs were normalized for body weight according to Hof [2].
**File 21_Fig_JointMoments_ slow.png:** represents the averaged 3D joint moments waveforms of right leg per age group (3–6 year, 7–8 year, 9–10 year, 11–12 year, >13 years) for walking at slow speed.
**File 22_Fig_JointPower_slow.png:** represents the averaged sagittal joint power waveforms of right leg per age group (3–6 year, 7–8 year, 9–10 year, 11–12 year, >13 years) for walking at slow speed.
**Folder 23_.mox files**: contains the .mox files for each child, organized per walking speed. The .mox files contain subject data (e.g. sex, body mass, knee and ankle width), marker position and force plate data, kinematic data (joint angles), kinetic data (GRF, joint moment, joint power) and EMG data (EMG is not described in this manuscript) generated by CAREN software (D-flow).
**Folder 24_.txt files:** contains .txt files for each child, organized per walking speed. The .txt files contain marker position and force data, kinematic data (joint angles), kinetic data (joint moment, GRF and joint power), EMG data and Center of Mass data generated by CAREN software (D-flow).
**Folder 25_xls files:** contains the processed data, including spatiotemporal parameters, joint angles, GRF, joint moment and joint power for each child, organized per walking speed. Data is generated by a private custom-made Matlab script for analyzing .mox data. Consider ‘26_Description_parameters’ for interpreting the data.
**File 26_Description_parameters:** contains information for interpreting .xls processed gait data. The layout and parameters are described in detail.

## 3 Experimental Design, Materials and Methods

### 3.1 The System

**Fig. 1:**
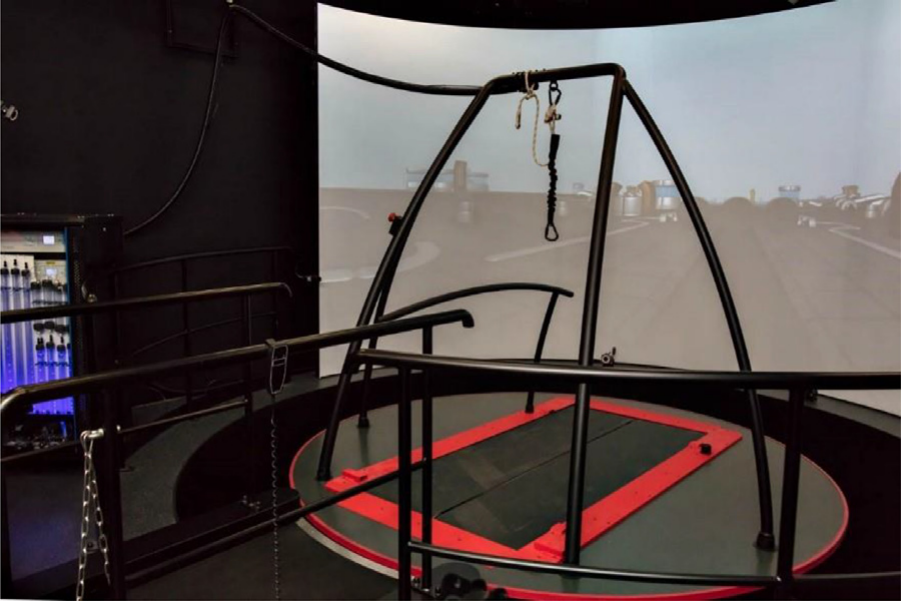
The Computer Assisted Rehabilitation ENvironment (CAREN) system.

Three dimensional (3D) gait analysis was performed at the Computer Assisted Rehabilitation Environment (CAREN, Motek Medical BV, Amsterdam, The Netherlands, Fig. 1). The CAREN system combines an instrumented split-belt treadmill (ForceLink, Culemborg, The Netherlands) with a 12-camera 3D motion capture system (VICON NEXUS v2.7, Oxford Metrics Group, Oxford, UK, 100 Hz).The individual treadmill belts have a length and width of 2.15 × 0.5 m, a motor of 6.28 kW per belt, a belt speed update frequency of 60 Hz, and a speed range of 0–18 km/h, 1000 Hz). The treadmill is integrated on a movable platform (flight simulator) and is surrounded by a 180° cylindrical screen in which a virtual environment is projected, resulting in optic flow that is synchronized with the walking speed. In addition, three two-dimensional cameras are used to record the sagittal and frontal view.

The hardware components of CAREN (e.g. treadmill, platform, projectors) are controlled by Motek software, specifically D-Flow control software (version 3.28.0). The 3D motion capture system is controlled by the Nexus software of Vicon (version 2.8.1).

### 3.2 System Preparation and Settings

Nexus 2.8.1 is used to calibrate the cameras and to zero level the force pates in preparation for the measurements. Camera calibration was done according to the dynamic calibration procedure embedded in Vicon, as described in the CAREN user manual (Motek Medical BV, December 8, 2021). Force threshold for step detection in Nexus was set at 25N.

In D-flow, the Gait Graphs_HBM2.caren application (walking in an industrial environment) is used for the gait measurement. For the inverse dynamics, a filter on marker and force plate data of 6Hz is used. The force plate configurations for the analog data was set at 15Hz for the low-pass prefilter frequency and 20N for the force threshold. All filters were unidirectional 2nd order Butterworth filters.

The Nexus and D-flow software are connected to each other. No markers or stick figure were visualized during the measurements.

### 3.3 Subject Preparation

First, a standardized physical examination according to Becher et al. was done to ensure that all included children had no physical impairments [3]. The physical examination includes body height (cm), weight (kg), leg length (cm; spina iliaca anterior superior to medial malleolus), active and passive range of motion of the hip, knee and ankle (°) and the assessment of position.

Afterwards, the comfortable walking speed was determined during overground walking trials. Children walked repeatedly over a 9 meter walkway at comfortable walking speed. The speed was measured using two movement detection ports placed 4 meters apart. At least five walking trials were used to compute the averaged comfortable walking speed.

**Fig. 2:**
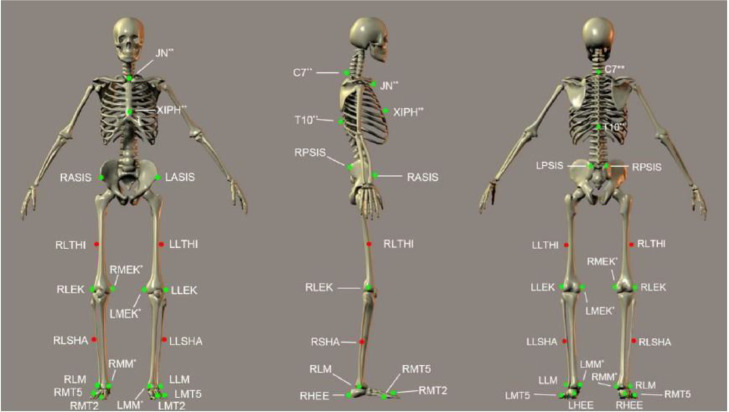
The Human Body Lower Limb Model with trunk markers (HBM2) [4].

Next, 26 reflective markers were placed at specific bony landmarks of the child according to the Human Body lower limb model with trunk markers (HBM2, Fig. 2 [4,5]) by two experienced operators (RS, RM).

After being secured to the system, a static (T-pose with feet at shoulder width and in parallel to the Z-axis of the coordinate system in D-flow each foot positioned at one belt and arms spread at shoulder height) and dynamic (walking a few steps) measurement was done in Nexus recording all reflective markers. The labeling model of Nexus was used to create the biomechanical model (HBM2). Next, a functional knee and hip calibration was performed in D-flow, which was also recorded in Nexus. For the functional knee calibration, the child had to flex and extend each knee about five times through a range of approximately 0-45°. For the functional hip calibration, the child was asked to perform one star arc movement per leg. The functional calibration is in line with the International Society of Biomechanics (ISB) guidelines [6]. Based on the functional calibration, the joint center of the hip and knee axis were determined [7]. Finally, a static subject calibration was done in D-flow, determining knee and ankle width and body weight.

Next, children walked for 6 min at comfortable walking speed (as determined by overground walking trials) at the treadmill to familiarize to the system. This is in line with Meyer et. al stating that six minutes of familiarization is needed to adapt a normal gait pattern [8].

### 3.4 Measurements

Each child walked at three walking speed conditions which were randomly applied in sequence: comfortable speed, slow speed (30% slower than comfortable) and fast speed (30% faster than comfortable). For each speed condition, 250 steps were recorded. Data was recorded in D-flow and Nexus.

Measurements were done while wearing standardized gym shoes and underwear to eliminate the effect of shoe wear and cloths. Moreover, a safety harness was worn to prevent falling.

### 3.5 Data Analysis

Data was first checked for quality using custom made scripts in Matlab (R2016a, Mathworks, Natick, MA, USA). The data quality check concerns for instance checking the visibility of markers. Gaps in marker detection (>30 samples) are identified and these strides are removed. Gaps <30 samples are interpolated using cubic spline fitting. In addition, force plate data is checked and strides were both feet hit one belt are excluded for kinetic analysis.

After quality check, step detection is done using custom made algorithms programmed in Matlab (R2016a, Mathworks, Natick, MA, USA). Step detection is based on a combination of heel marker kinematics (lowest point of that marker during gait) and force plate data (exceeding the threshold of 50N) as described by Zeni et al. [9].

Custom-made Matlab scripts programmed in Matlab (R2016a, Mathworks, Natick, MA, USA), relying on published formulas and principles, were used to calculate spatiotemporal parameters, joint angles, GRFs, joint moments and joint power.

### 3.6 Outcomes

The following parameters were calculated:

- Spatiotemporal parameters such as step length (m), step time (s).
- Joint angles of trunk, pelvis, hip, knee and ankle in sagittal, frontal and transversal plane, presented as a function of the gait cycle. Data is time normalized with 0% representing initial contact and 100% representing next initial contact of the same leg.
- Vertical and anterior-posterior GRF presented as a function of the gait cycle. Data is time normalized with 0% representing initial contact and 100% representing next initial contact of the same leg.
- Joint moments of hip, knee and ankle in sagittal, frontal and transversal plane, presented as a function of the gait cycle. Data is time normalized with 0% representing initial contact and 100% representing next initial contact of the same leg.
- Sagittal joint power of hip, knee and ankle, presented as a function of the gait cycle. Data is time normalized with 0% representing initial contact and 100% representing next initial contact of the same leg.

Data are provided for each child separately and per walking speed. Data of every stride of both legs is presented. In addition the averaged gait pattern of each child, calculated as the average parameter of all valid individual steps, is presented. Moreover data is presented and visualized per age group.

## Ethics Statements

All parents and children aged 12 years and older provided written informed consent prior to participation. The protocol was approved by the local Medical Ethics Committee of the MUMC+ (NL51929.068.14/METC142082).

## CRediT authorship contribution statement

**Rachel Senden:** Conceptualization, Methodology, Validation, Formal analysis, Investigation, Resources, Writing – original draft, Writing – review & editing, Visualization, Project administration. **Rik Marcellis:** Conceptualization, Methodology, Validation, Formal analysis, Investigation, Resources, Writing – review & editing, Project administration. **Kenneth Meijer:** Conceptualization, Methodology, Writing – review & editing. **Paul Willems:** Software, Validation, Writing – review & editing. **Ton Lenssen:** Writing – review & editing. **Heleen Staal:** Writing – review & editing. **Yvonne Janssen:** Investigation, Writing – review & editing. **Vincent Groen:** Investigation, Writing – review & editing, Project administration. **Roland Jeroen Vermeulen:** Conceptualization, Methodology, Writing – review & editing. **Marianne Witlox:** Conceptualization, Methodology, Resources, Writing – review & editing, Project administration.

## Declaration of Competing Interest

The authors declare that they have no known competing financial interests or personal relationships that could have appeared to influence the work reported in this paper.

## Data Availability

Dataset of 3D gait analysis in typically developing children (Original data) (OSF).
